# The life cycle of a zoonotic parasite reassessed: Experimental infection of *Melanoides tuberculata* (Mollusca: Thiaridae) with *Centrocestus formosanus* (Trematoda: Heterophyidae)

**DOI:** 10.1371/journal.pone.0194161

**Published:** 2018-04-06

**Authors:** Hudson A. Pinto, Nicole Q. Gonçalves, Danimar López-Hernandez, Eduardo A. Pulido-Murillo, Alan L. Melo

**Affiliations:** Department of Parasitology, Institute of Biological Sciences, Universidade Federal de Minas Gerais, Belo Horizonte, Minas Gerais, Brazil; Charles University, CZECH REPUBLIC

## Abstract

*Centrocestus formosanus* is a foodborne intestinal trematode that is native to Asia and has been introduced into the Americas and Europe. Although there are several studies of *C*. *formosanus* in definitive vertebrate hosts (birds and mammals, including humans), and in intermediate vertebrate hosts (fish and amphibians), there is little published information regarding interaction with its transmitting mollusc. In this study we studied the miracidial development of *C*. *formosanus* using a mouse as a source of eggs. Adult parasites were maintained in water in order to develop miracidia in intrauterine eggs. Miracidia appeared at 12 days of incubation, with no hatching observed for up to 40 days. Subsequently, we placed dead *C*. *formosanus* containing eggs with miracidia individually in contact with 48 specimens of *Melanoides tuberculata*, and observed the absence of the parasites after 1h of exposure, suggesting that they were ingested by the snails. Of the 33 experimentally-infected snails that were alive after 84–89 days post-infection (DPI), seven (21%) shed cercariae. We detected young *C*. *formosanus* rediae in 21/33 (64%) *M*. *tuberculata* at 90 DPI. To our knowledge, this report is the first to show that, in the life cycle of *C*. *formosanus*, infection of molluscs occurs passively by ingestion of eggs, followed by a long intramolluscan phase. We compare these data with those described for other Heterophyidae, and discuss on the phylogenetic background of the pattern of miracidial development verified in these parasites.

## Introduction

*Centrocestus formosanus* (Nishigori, 1924) is an intestinal foodborne trematode that parasitizes birds and mammals, including humans. The species is native to Asia, and was introduced in the Americas and Europe [1–4]. The worldwide expansion of *C*. *formosanus* probably is related to the wide distribution of its principal molluscan intermediate host, the invasive thiarid *Melanoides tuberculata* (Müller, 1774), which has been reported to be naturally infected with this heterophyid in more than 10 countries, both in Asia and the Americas [5]. Fish are the second intermediate hosts of *C*. *formosanus*, and dozens of species have been reported to be naturally infected worldwide [1, 6–11]. Metacercariae encysted in gills cause pathological alterations related to developmental delay and death, giving rise to economic losses in the fish farming industry [12].

Recent taxonomic studies report the appearance of *C*. *formosanus* in new areas and hosts, including new human cases [2, 7, 13–15]. In addition, new studies have reported the interaction of *C*. *formosanus* with various hosts, both naturally-infected snails [16] and experimentally-infected fish, amphibians and rodents [17–20]. Moreover, several studies related to treatment, control, and monitoring strategies for aquatic ecosystems were performed in the last several years [21–24]. Moreover, molecular studies relating to identification and phylogenetic inference have been published recently [11, 25]. Despite these advances, details regarding the development of *C*. *formosanus* in its first intermediate host are poorly understood.

According to Nishigori (1924) [3], the miracidia of *C*. *formosanus* hatch 15 days after incubation of eggs in water. This fact has been disputed, since it suggests that snail infection occurs actively by penetration of miracidia. This life cycle pattern would be different from that of almost all members of the superfamily Opisthorchioidea, in which infection of the snail takes place after ingestion of eggs, with hatching of miracidium occurring within the digestive tract [26]. Therefore, it is necessary to reassess the development of miracidia of *C*. *formosanus* and the mechanism of infection of the molluscs. In addition, the time of infection (the pre-patent period) and the degree of susceptibility of *M*. *tuberculata* to *C*. *formosanus* are currently unknown. In the present study, we evaluated these biological parameters related to the life cycle of *C*. *formosanus*. We then compared our data with that of other known members of the family Heterophyidae.

## Material and methods

### Experimental infection of vertebrate hosts (fish and mouse)

We performed experimental infections using cercariae that had emerged from one specimen of naturally-infected *M*. *tuberculata* in Brazil. We used pleurolophocercous cercariae that had emerged from *M*. *tuberculata* after artificial photostimulation to infect 30 specimens of laboratory-reared *Poecilia reticulata* Peters, 1859. We placed fish individually in six-well plates containing cercarial solution with approximately 300 larvae. Fish were left in contact with cercariae for 2 hours and were then transferred to a plastic recipient containing about 2 L water, where they were fed daily. Fish were maintained for 30 days, at which time they were euthanized with benzocaine hydrochloride solution > 250 mg/L. The gills were removed and examined under a light microscope in order to count metacercariae.

Fragments of the gills containing approximately 200 metacercariae of *C*. *formosanus* were administered orally to one Swiss mouse. We treated the mouse daily with dexamethasone sodium phosphate (50 mg/Kg, s.c.) in order to increase the number of parasites recovered [20]. At 12 days post-infection (DPI), the mouse was euthanized by barbiturate overdose, and the small intestine was transferred to a Petri dish containing saline (0.85% NaCl). The small intestine was opened longitudinally and was examined under a stereomicroscope. Adult parasites found in the proximal third were collected with a micropipette and were maintained in saline until analysis.

This study was carried out in accordance with the recommendations for the care and use of laboratory animals, and was approved by the Committee on the Ethics of Animal Experiments of the Universidade Federal de Minas Gerais (CEUA UFMG protocol 20/2016).

### Parasite identification

Samples of cercariae, metacercariae and adult parasites were studied alive by light microscopy. We observed general traits characteristic of *C*. *formosanus* and compared them to the description of various authors [2,27,28]. Ten parasites were fixed in ethanol and were used for molecular identification by amplification and sequencing of regions of the 18S rDNA. For this, we extracted DNA using the Wizard Genomic DNA Purification kit (Promega, USA) and subjected samples to PCR. The primers and conditions of PCR were as described by Moszczynska *et al* (2009) [29]. The sequences obtained (561bp) were compared to those in the GenBank public sequence database using the Basic Local Alignment Search Tool (BLAST).

### Miracidial development

To evaluate the development of miracidia in intrauterine eggs, adult parasites (n = 120) recovered from the mouse were transferred to tubes containing 2 mL of distilled water. This procedure killed the parasites, but did not interfere with the viability of the eggs. Subsamples of 5 dead parasites were examined by light microscopy twice per week for 40 days for evaluation of the development and vitality of miracidia. We removed parasite samples from the tubes with a micropipette and mounted them on glass slides with coverslips. All eggs present in the uterus of each parasite were visualized under high magnification (1000 x) by light microscopy. We performed morphological and morphometric studies using a Leica DM500 microscope connected with a Leica ICC50 HD digital camera. We determined the vitality of miracidia by visualization of their morphological integrity and movement inside embryonated eggs. For each exam, we recorded the presence of free miracidia or empty eggshells. Each tube was subjected to slow centrifugation and the water was changed.

### Experimental infection of *Melanoides tuberculata*

We used *M*. *tuberculata* measuring 7–10 mm for experimental infection. The snails were collected at a body of water located in the municipality of Lagoa Santa (19°37'44"S, 43°54'80"W), state of Minas Gerais, southeastern Brazil and the prevalence of infection of *M*. *tuberculata* with *C*. *formosanus* was less than 0.5%. The snails were maintained under laboratory conditions for about 2 months and were negative for trematode infection as assessed by photostimulation. As the source for infection, we used a subsample of dead *C*. *formosanus* previously submitted to study of miracidial development. These dead parasites were previously incubated in distillated water for 40 days and presented embryonated eggs. Specimens of *M*. *tuberculata* (n = 48) were individually transferred to 24-well culture plates containing about 2 ml of chlorine-free water, along with one dead adult parasite containing embryonated eggs. After about 1 h of exposure, the plates were evaluated under a stereomicroscope to detect if the added dead parasites had been consumed by the snails. The molluscs were transferred to a plastic receptacle containing 1 L of chlorine free water (renewed weekly) where they were fed with lettuce leaves. A group of *M*. *tuberculata* (n = 70) not exposed to parasite eggs was maintained under the same conditions as the control group. Every week, starting at 30 DPI, we evaluated mollusc mortality and success of infection. We used the Chi-square test to calculate differences in mortality between the group of *M*. *tuberculata* experimentally infected with *C*. *formosanus* and the control group, and difference was considered statistically significant if *P* < 0.05. The emergence of cercariae from the experimentally infected snails were verified after 2 hours of artificial photostimulation followed by examination under a stereomicroscope. The number of cercariae emerging from each snail was counted in the first days after the detection of positive snails. To rule out the possibility of natural infection with *C*. *formosanus*, the control group was also subjected to the photostimulation test.

At 90 DPI, all snails exposed to dead parasites but negative for cercarial emergence were crushed between glass plates and examined under stereomicroscope for the presence of cercariae and rediae of *C*. *formosanus*. This procedure was also performed on the control group. The snails were maintained at room temperature (18–27°C) under daily photoperiods of 12 hours of light and 12 hours of dark. The experiment was conducted from November 10 2016 to February 13 2017 and the temperature reported for the locality in this period ranged from 19°C to 31°C, corresponding to the hot season.

## Results

### Parasite identification

The following morphological features observed in the material examined for this study were in accordance with previous descriptions of the developmental stages of *C*. *formosanus*: cercariae characterized as pleurolophocercous type; oval-shaped metacercariae presenting an X-shaped excretory vesicle with dark granules inside; small adults with a double crown of 32 circumoral spines, entire ovary, two opposite posterior testes, follicular vitellaria extending laterally along the body and few eggs in the uterus [2, 4, 6, 9, 10, 27, 28]. We corroborated the identification by amplification and sequencing of a partial fragment of the 18S rDNA, revealing 99.8% and 100% similarity with isolates of *C*. *formosanus* from USA (AY245759) and Thailand (HQ874608), respectively. The sequence of the Brazilian isolate of *C*. *formosanus* was deposited at GenBank under the accession number MG778684.

### Miracidial development

**Fig 1:**
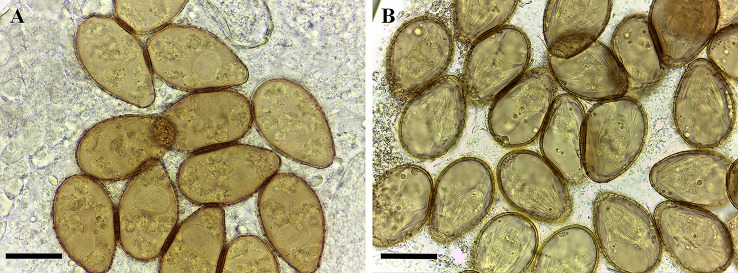
Intrauterine eggs of *Centrocestus formosanus*. **(A)** Non-embryonated (immature) and **(B)** embryonated after 12 days of incubation. Scale bars = 20 μm.

Eggs (n = 20) observed in the uteri of live parasites were 35 ± 2 (33–39) μm long and 19 ± 1 (18–21) μm wide. They were immature (non-embryonated, as verified in the feces of the definitive host) with a zygotic cell (single cell stage) measuring 10 ± 1 μm in diameter, surrounded by granular vitelline substance (Fig 1A). Incubation of dead adult parasites in distilled water resulted in formation of miracidia inside intrauterine eggs starting at the 12th day of incubation (Fig 1B). Structural degradation of the parasites was observed throughout the period of study, which did not interfere in the miracidial development and viability. Miracidia (n = 20) measured 23 ± 3 μm in length and 12 ±1.5 μm in width, with cilia and a cephalic hyaline gland in their interior. No other details of miracidia morphology were visualized. The vitality of miracidia was verified throughout the experiment by observing the morphological integrity and movements of these larvae. The lattice design of the egg shell surface remained unaltered after incubation. We did not observe miracidia hatching at up to 40 days of incubation. Moreover, no empty shell that could suggest miracidia hatching was verified during the exams of intrauterine eggs. All eggs evaluated were embryonated.

### Experimental infection of *Melanoides tuberculata*

**Fig 2:**
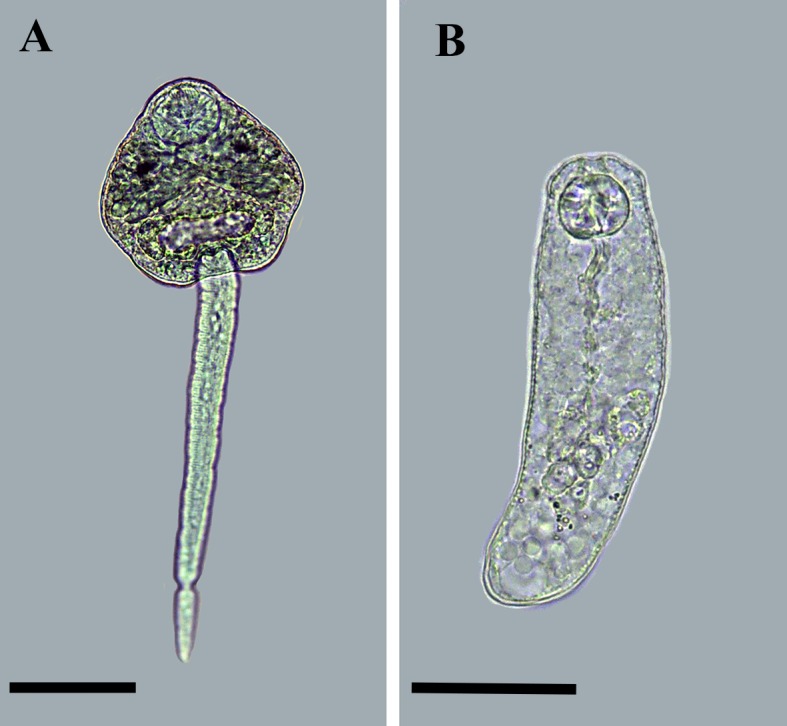
Larval stages of *Centrocestus formosanus* found in experimentally infected *Melanoides tuberculata*. **(A)** Cercaria obtained after photostimulation test performed at 84 days after infection. **(B)** Young rediae found in mollusc evaluated by the crushing method at 90 days of infection. Scale bars: 50 μm.

Evidence of ingestion of the infective stages of *C*. *formosanus* by *M*. *tuberculata* was obtained because not a single dead specimen added in the culture plates with the snail was found after 1hr of contact between them. Of 48 specimens of *M*. *tuberculata* exposed to dead parasites with mature eggs, 33 (69%) survived until 90 DPI. The survival rate in the control group was similar [(51/70); 73%, χ2 = 0.2342, *P* = 0.63)]. In the experimental group, 7/33 (21%) snails shed *C*. *formosanus* cercariae after photostimulation (Fig 2A). We detected infected snails at 84–89 DPI. In the first days after the detection of infection, we verified the emergence of few cercariae after 2 hours of photostimulation. Six of the positive snails shed few cercariae (1–14) in the initial phase. The emergence of cercariae was not constant, and several of the snails previously detected as positive were negative in some subsequent exams, revealing that, at least in the initial phase of the infection, the release of cercariae did not occur daily. An exception to this pattern was one specimen that shed 135 (39–322) cercariae, and was detected positive every day of analysis. The specimens of *M*. *tuberculata* from the experimental group negative for cercariae after the photostimulation test were crushed at 91 DPI, and 21/26 (81%) were positive for immature rediae. These rediae (n = 10) measured 140 ± 5 μm by 40 ± 4 μm, with pharynx measuring 19 ± 2 μm by 20 ± 1 μm. The caecum of these young rediae was slender, elongated, with a globular terminal portion, extending up to the posterior third of the body.

Considering together the data obtained by shedding and crushing methods of analysis of infection, the rate of experimental infection of *M*. *tuberculata* with *C*. *formosanus* was 85% (28/33). All snails from the control group were subject to artificial photostimulation, but no cercariae were found. These snails were crushed at the end of the experiment and no specimens were found infected with larval stages of *C*. *formosanus* or other trematodes.

## Discussion

*Centrocestus formosanus* was originally described in what is today Taiwan by Nishigori (1924) [3]. Various aspects of its life cycle were described by Nishigori as follows: (1) molluscs of the family Thiaridae (identified as a species of *Melania*) are the first intermediate hosts; (2) pleurolophocercous cercariae emerge from the snails; (3) fish are second intermediate hosts, (4) birds and mammals, including humans (Nishigori infected himself) are the definitive hosts; (5) unembryonated eggs develop in the environment, forming miracidia, which then hatch and develop directly to rediae after actively infecting the mollusc. Two decades later, most of the details of the life cycle of *C*. *formosanus* described above (except 5) were corroborated by Chen (1942, 1948) [27, 28], who presented a more detailed description of some larval stages, and described new naturally-and experimentally-infected hosts in China. Chen highlighted that the omission of the sporocyst stage described by Nishigori [3] was different from the pattern of life cycle of some heterophyids known at that time [28]. A few years later, the introduction of *C*. *formosanus* onto the American continent was reported by Martin (1958) [30], who found the parasite infecting a mollusc identified at the time as *Stenomelania newcombi* (Lea, 1856), currently considered a probable misidentification of *M*. *tuberculata* [7]. Martin described new morphological and experimental data regarding larval stages of *C*. *formosanus*, including the presence of elongated sporocysts in naturally-infected snails [30]. Despite these descriptions, experimental evidence regarding the form of infection (active or passive) of the snail by *C*. *formosanus* has been lacking to date. In addition, the developmental period of the parasite in its intermediate host has remained unknown.

In the present study, we corroborated observations made by Nishigori (1924) [3] regarding the development of the miracidium of *C*. *formosanus* in the external environment. The developmental period for miracidia in eggs that we reported here (12 days) was similar to the 15 days reported by Nishigori (1924) [3]. Regarding the formation of miracidia in representatives of the family Heterophyidae, two processes were described: the release of embryonated eggs by the parasite, and the formation of miracidia in the environment. The first phenomenon was the most frequent, as can be verified in species belonging to the genera *Ascocotyle* [31–33], *Haplorchis* [34, 35], *Heterophyes* [36], *Metagonimus* [37, 38], *Pygidiopsis* [39, 40], *Stellantchasmus* [41] and *Stictodora* [42]. On the other hand, release of immature eggs that embryonate in the external environment, after incubation for 10–23 days, depends on the species. This has been described for *Acetodextra amiuri* (Stafford, 1900) (14 days) [43], *C*. *formosanus* (12–15days) [3, present study], *Cryptocotyle lingua* (Creplin, 1825) (10 days) [44], *Cryptocotyle concava* (Creplin, 1825) (embryonation not evaluated) [45], *Euryhelmis monorchis* Ameel, 1938 (embryonation not evaluated) [46] and *Metagonimoides oregonensis* Price, 1931 (23 days) [47]. Interestingly, in two genera of heterophyids, the occurrence of both patterns of egg development was verified. In the genus *Apophallus*, the species *Apophallus donicus* (Skrjabin and Lindtrop, 1919) and *Apophallus muehlingi* (Jagerskiold, 1899) produce embryonated eggs [48, 49], whereas in *Apophalus imparetor* Lyster, 1940 and *Apophalus venustus* (Ransom, 1920), the miracidium is formed after 14 and 18 days of incubation in the external environment, respectively [50, 51]. Similarly, in *Haplorchoides cahirinus* (Looss, 1896) and *Haplorchoides mehrai* Pande and Shukla, 1976, eggs are released mature [52, 53], while in *Haplorchoides vacha* Agrawal and Agrawal1981, the formation of the larva occurs after 9 days of incubation of the eggs [54]. In these cases, a revision of the parameters or confirmation of the correct identification of the parasites may be necessary, since occurrence of both processes of egg development in a same natural genus appears unlikely. Considering representatives of members of the superfamily Opisthorchioidea, the release of immature eggs was described for some cryptogonimids, such as *Aphalloides coelomicola* Dollfus, Chabaud and Golvan, 1957 [55, 56] and *Stemmatostoma pearsoni* Cribb, 1986 [57]. Regarding Opisthorchiidae, to the best of our knowledge, all species produce embryonated eggs.

Recently, molecular studies have revealed new insights regarding the phylogeny of members of the superfamily Opisthorchioidea, including the paraphyly of Heterophyidae and an uncertain position of *C*. *formosanus* [55, 58–60]. Future studies may suggest a new arrangement of the group. Such studies will certainly be more robust if they consider biological aspects related to the members of this superfamily. The extent to which there is a phylogenetic signal in the degree of development of related eggs in species of the Heterophyidae is an open question and requires further examination. For example, it is interesting to note that the grouping of the genera *Euryhelmis*, *Cryptocotyle* and *Metagonimoides*, which produce non-embryonated eggs, was recently verified [55]. The affinity between the two first genera was also reported using different markers [58,60]. On the other hand, the main clade containing species of Heterophyidae verified by these authors are formed by species that produce embryonated eggs (genera *Haplorchis*, *Haplorchoides*, *Procerovum*, *Stellantchasmus* and *Metagonimus*) [58, 60]. Regarding *C*. *formosanus*, its affinity with some members of Cryptogonomidae or a basal position in Opisthorchioidea has been demonstrated [60]. Interestingly, the partial 18S sequences obtained in our study were 98.6% similar to *A*. *amiuri* (KM538122) and *C*. *lingua* (AJ287492), two species that also produce unembryonated eggs [43, 44].

In the present study, no hatching of miracidium was observed after 40 days of incubation of eggs of *C*. *formosanus*, unlike what was reported by Nishigori (1924) [3]. The factors related to this difference are difficult to conceptualize. However, it is important to mention that the possibility of transmission of trematodes to molluscs by the ingestion of eggs (without hatching of miracidium in the external environment) was described for the first time by Faust and Khaw (1927) [61] in the study on the life cycle of *Clonorchis sinensis* (Cobbold, 1875), a member of the family Opisthorchiidae. There is a consensus that, in members of the superfamily Opisthorchioidea, hatching of miracidia occurs in the intestinal tract of the snails. In fact, the absence of spontaneous miracidial hatching was reported for several species of Heterophyidae [43–45, 47, 51]. The only species of Heterophyidae for which miracidial hatching was described to occur in the external environment are *C*. *formosanus* [3] and *H*. *vacha* [54]. Interestingly, the spontaneous hatching of miracidia was not reported for two other species of *Haplorchoides* (*H*. *cahirinus* and *H*. *mehrai*) [52,53]. Therefore, a reassessment of the life cycle of *H*. *vacha* is also necessary.

The results here obtained by experimental infection of *M*. *tuberculata* with *C*. *formosanus* confirm that the transmission of the parasite to the mollusc occurs by ingestion of fully embryonated eggs, as already verified for other heterophyids. The prepatent period we verified in *M*. *tuberculata* experimentally infected with *C*. *formosanus* was 84 days. However, most molluscs found negative after photostimulation exam presented immature rediae of *C*. *formosanus*. This finding suggests that a time greater than 90 days may be necessary for the emergence of the first cercariae from this parasite. Compared to some species of the family, this time span of intramolluscan development may be considered long, given that prepatent periods equal to or less than 60 days have been reported in snails experimentally infected with *Pygidiopsis macrostomum* Travassos, 1928 [40], *Ascocotyle longa* Ransom, 1920 [33], *Haplorchis yokogawai* (Katsuta, 1932) [34], and *H*. *cahirinus* [52]. On the other hand, pre-patent periods longer than those reported here for *C*. *formosanus* have been described for *Ascocotyle tertia* Ostrowski de Núñez, 2001 (120 DPI) [32] and *Stellantchasmus falcatus* Onji and Nishio, 1916 (123 DPI) [41]. Yet, the temperature or season of the year can influence the development time for trematodes in snails, as verified for *P*. *macrostomum*, where development was longer in the winter (56 DPI) than in the summer (34 DPI) [44]. In the present study, experimental infection was performed during the hot season (18–27°C), suggesting that the time of development of *C*. *formosanus* in *M*. *tuberculata* in lower temperatures might be greater than 90 days. Although this time may be considered too long, it is important to mention that the life span reported to *M*. *tuberculata* is 2 to 3.5 years [62].

The number of *C*. *formosanus* cercariae that emerged from the seven experimentally-infected *M*. *tuberculata* specimens might be considered small. This is probably because the experimentally-infected snails were in the early stage of infection. The number of cercariae observed in naturally-infected molluscs can be very variable, and may be influenced by various factors. An extremely large amount of *C*. *formosanus* cercariae (averaging more than 1600 cercariae/24 h, but up to 63,400 larvae/24 h) was described in naturally-infected snails [63]. In our experience, in evaluation of naturally-infected *M*. *tuberculata* from Brazil (exposed to photostimulation for about 2 hours), the number of cercariae is frequently larger than the number we observed in experimentally-infected snails in this study. This difference is possibly due to the fact that our snails were in the initial phase of production of cercariae, which can be confirmed by further study. Data obtained in the present study suggest that approximately 90 days or more are necessary as a quarantine time to rule-out natural infection with *C*. *formosanus*. Although, our quarantine time (60 days) was less than the ideal, the maintenance and analysis of an unexposed control group, which were negative for infection with trematodes at the end of the experiments, support the data we obtained for the group of *M*. *tuberculata* experimentally infected with *C*. *formosanus*.

To date, there have been few studies evaluating experimental infection of molluscs with species of the family Heterophyidae. The initial phase between egg ingestion by the snail and the production of cercariae in its digestive glands is unknown for almost all known species of heterophyids. Most data on intramolluscan development is based on analysis of developmental stages recovered from naturally-infected snails [30, 64–69]. Difficulties related to the rearing and maintenance of potential intermediate hosts in laboratory conditions preclude experimental infection studies involving this group of parasites. In fact, the high specificity for the first intermediate host known for most trematode species, including heterophyids, makes it difficult to perform experimental infection studies in snails with parasite eggs obtained from a naturally-infected vertebrate. Moreover, possible difficulties related to separation and manipulation of extremely small eggs, or undetected amounts in the feces of experimental models, as verified in *Centrocestus* spp. [27, 70] may be a hindrance to conducting such studies. In this sense, the use of intrauterine eggs found in adult parasites, as performed in the present study, is an approach that facilitates experimental snail infection with heterophyids and closely-related trematodes. Previous studies used crushed parasites as source of eggs [32, 41]. In the present study we used whole parasites, which facilitated individual infection of snails as well as certification of ingestion of infective stages by all animals. However, we believe that the relatively small number of intrauterine eggs verified in *C*. *formosanus* [2, 20, 27] validates the use of the experimental protocol used in this study. It is possible that, in other parasites, the amount of intrauterine eggs was too high and lethal to the snails. Evaluation of this last possibility will require future studies. In natural conditions, the ingestion of eggs from the parasites eliminated in the host feces is probably the most important mechanism for infection of molluscs. However, we believe that the ingestion of parasites eliminated in the feces may also be possible, though it has not yet been discussed in the context of the biology of trematodes. The complete life cycle of *C*. *formosanus* was here reproduced under laboratory conditions for the first time. Considering the period of development and maturity of larval and adult stages in the various parasite hosts, it can be estimated that at least 4 months are required for the complete course of the life cycle of *C*. *formosanus* under laboratory conditions (at least 84 days for intramolluscan development, 20 days in the fish, 7 days for production of eggs by adult parasites, and 12 days for development of miracidium in the eggs). The new information on the experimental infection of snails with *C*. *formosanus* here presented may stimulate further studies on the asexual development of parasite, including the mortality rate for molluscs or the development of morphological abnormalities (e.g. gigantism, nanism), and physiological changes (e.g. parasitic castration) caused by *C*. *formosanus* in *M*. *tuberculata*.

## Conclusion

For the first time, we report experimental infection of *M*. *tuberculata* with *C*. *formosanus*. Data obtained revealed that the infection of the snails occurs passively by the ingestion of eggs embryonated in the external environment, following a pattern common to related species. We demonstrated that *M*. *tuberculata* is highly susceptible to *C*. *formosanus* and the time span of intramolluscan development of this heterophyid is about 90 days and at least 4 months are required for the complete course of the life cycle of the parasite under laboratory conditions.
